# Efficacy and safety of short-course antibiotic therapy for community-acquired pneumonia in adults: a meta-analysis

**DOI:** 10.3389/fmed.2026.1861027

**Published:** 2026-07-15

**Authors:** Ying Li, Songhe Zhao, Wenhua Su

**Affiliations:** 1 Department of Intensive Care Medicine 1, Jinan Third People's Hospital, Jinan, Shandong, China; 2 Department of General Practice, Yunpu Street Community Health Service Center of Huangpu District, Guangzhou, Guangdong, China

**Keywords:** antibiotic therapy, antimicrobial stewardship, community-acquired pneumonia, meta-analysis, systematic review, treatment duration

## Abstract

**Background:**

Optimizing antibiotic duration for community-acquired pneumonia (CAP) is a critical priority for global antimicrobial stewardship. While short-course therapies (≤5 days) show clinical promise, robust, updated evidence comparing their specific safety profiles, particularly regarding severe adverse events, against traditional long-course regimens (>5 days) is required to reliably guide clinical practice.

**Methods:**

Following the PRISMA 2020 guidelines (PROSPERO: CRD420261278947), we systematically searched PubMed, Embase, and CENTRAL up to January 15, 2026, for randomized controlled trials (RCTs) comparing short-course (≤5 days) and long-course (>5 days) antibiotic therapies in adults with CAP. The primary outcome was clinical success. Secondary outcomes included bacteriological and radiological success, mortality, treatment discontinuation, and a detailed profile of adverse events (AEs).

**Results:**

A total of 21 RCTs were included. Short-course antibiotic therapy achieved comparable efficacy to long-course therapy in clinical success (RR = 1.00, 95% CI: 0.98–1.02, *p* = 0.77), bacteriological success (RR = 1.00, 95% CI: 0.97–1.03, *p* = 0.86), radiological success (RR = 1.03, 95% CI: 0.97–1.09, *p* = 0.29), all-cause mortality (RR = 0.79, 95% CI: 0.47–1.32, *p* = 0.37), and treatment discontinuation (RR = 0.95, 95% CI: 0.68–1.31, *p* = 0.74). While short-course regimens were associated with a marginally higher risk of mild treatment-related AEs (RR = 1.12, 95% CI: 1.00–1.26, *p* = 0.04), they showed a non-significant downward trend in treatment-related serious adverse events (SAEs) (RR = 0.37, 95% CI: 0.13–1.07, *p* = 0.07). Most included studies demonstrated generally acceptable methodological quality with an overall low risk of bias.

**Conclusion:**

Short-course antibiotic therapy yields comparable clinical, bacteriological, and radiological efficacy for adults with CAP, with a favorable safety trend for severe adverse events. By maintaining equivalent efficacy while reducing cumulative antibiotic exposure, this regimen aligns with global antimicrobial stewardship priorities and provides robust evidence to support shortened antibiotic duration as a standard approach for appropriately selected adult CAP patients.

**Systematic review registration:**

https://www.crd.york.ac.uk/PROSPERO/view/CRD420261278947, identifier CRD420261278947.

## Introduction

Community-acquired pneumonia (CAP) remains a leading cause of infectious disease-related morbidity and mortality worldwide, imposing a profound clinical and economic burden on healthcare systems (1). Despite advancements in diagnostic precision and supportive care, the management of CAP is frequently complicated by the necessity of empirical antibiotic therapy (2, 3). While clinical guidelines provide frameworks for initial antimicrobial selection, the optimal duration of therapy, the balance point at which therapeutic benefit is maximized while minimizing antibiotic-related harms, remains a subject of intense clinical debate (4).

Historically, treatment courses often extended to 7–14 days, a paradigm driven more by clinical convention than by rigorous scientific evidence. Today, prolonged antibiotic exposure is recognized as a primary driver for the emergence of antimicrobial resistance (AMR), one of the most urgent global public health threats of the 21st century (5). Furthermore, extended courses increase the risk of adverse drug events, such as *Clostridioides difficile* infections, disrupt the host microbiome, and escalate healthcare expenditures (6). Consequently, optimizing and shortening treatment duration is now a core mandate of modern antimicrobial stewardship programs, aiming to maximize clinical efficacy while mitigating ecological and patient-level harms.

Recent clinical paradigms have increasingly favored shorter antibiotic courses. Several landmark randomized controlled trials (RCTs) have demonstrated that short-course therapy (e.g., 3 to 5 days) achieves comparable efficacy to traditional prolonged regimens in adult patients with CAP (7, 8). Despite these promising developments, the evidence comparing short-course (≤5 days) versus conventional long-course (>5 days) antibiotic regimens remains fragmented across trials with varying designs, diverse geographic populations, and differing antibiotic classes. Prior systematic reviews have not fully incorporated the most recently published trials, including those evaluating novel antimicrobial agents, and individual RCTs are often statistically underpowered to definitively detect nuanced differences in secondary outcomes, particularly regarding specific adverse events (AEs), severe adverse events (SAEs), and mortality. This creates a persistent knowledge gap for clinicians striving to implement duration-optimized protocols with confidence.

To address this gap and provide an up-to-date synthesis of the highest-quality evidence, we conducted a comprehensive systematic review and meta-analysis of RCTs comparing short-course (≤5 days) versus long-course (>5 days) antibiotic therapy in adults with CAP. The primary objective was to evaluate clinical success at the test-of-cure visit. Crucially, we also sought to conduct a granular analysis of secondary endpoints, including total and treatment-related adverse events, serious adverse events, bacteriological and radiological success, mortality, and treatment discontinuation. Prespecified subgroup analyses stratified by antibiotic class and sensitivity analyses were further performed to explore sources of heterogeneity and verify the robustness of pooled estimates, to provide a definitive, quantitative summary that informs daily clinical practice and future antimicrobial stewardship guidelines.

## Method

### Data sources and searches

This systematic review and meta-analysis was conducted in accordance with the Preferred Reporting Items for Systematic Reviews and Meta-Analyses (PRISMA) 2020 statement. The review protocol was prospectively registered with the International Prospective Register of Systematic Reviews (PROSPERO) (registration number: CRD420261278947). We systematically searched PubMed, Embase, and the Cochrane Central Register of Controlled Trials (CENTRAL) from database inception up to January 15, 2026. A comprehensive Boolean search strategy was employed, utilizing a combination of key terms and medical subject headings related to CAP, antibiotic treatment, treatment duration, and RCTs. Furthermore, this electronic search was supplemented by manually screening the reference lists of relevant systematic reviews and included trials to identify any additional eligible titles.

### Study selection

Study eligibility was determined based on the following pre-defined inclusion criteria: (i) Study design: RCTs; (ii) Population: adults (aged ≥18 years) with a confirmed diagnosis of CAP, defined by integrated clinical, radiological, and microbiological findings; (iii) Intervention and Comparison: randomized allocation to treatment arms comparing short-course (≤5 days) versus long-course (>5 days) antibiotic therapy; and (iv) Outcomes: reporting of relevant clinical outcomes, including but not limited to clinical success rates, adverse events, treatment discontinuation, and mortality.

Studies were excluded if they met any of the following criteria: (i) inclusion of patients with significant confounding comorbidities that could influence treatment outcomes; (ii) non-randomized designs, including observational, cohort, and case–control studies; or (iii) unpublished data, non-peer-reviewed articles, or studies without accessible full texts; (iv) not published in the English language.

The study selection followed a standardized two-stage independent screening process. Initially, two reviewers (Y.L. and S.Z.) independently screened all retrieved titles and abstracts to identify potentially eligible articles. Subsequently, the full texts of all candidate articles were retrieved and independently evaluated by the same two reviewers to determine final eligibility. All exclusion decisions were cross-verified by the second reviewer to minimize selection bias. Discrepancies at either screening stage were resolved through discussion to reach a consensus, or via consultation with a third reviewer (W.S.), who served as the final arbiter.

### Outcome measures

The primary outcome was clinical success (defined as clinical cure or significant improvement) at the test-of-cure (TOC) visit, characterized by the resolution or marked improvement of the baseline signs and symptoms of CAP without the requirement for additional antibiotic therapy.

Secondary outcomes included the following: (i) adverse event profiles, including total adverse events (AEs), treatment-related AEs, total serious adverse events (SAEs), and treatment-related SAEs; (ii) bacteriological success, defined as the documented or presumed eradication of the baseline pathogen; (iii) radiological success, defined as the improvement or complete resolution of pulmonary infiltrates on chest imaging; (iv) all-cause mortality; and (v) treatment discontinuation.

### Data extraction and quality assessment

Two reviewers (Y.L. and S.Z.) independently conducted data extraction and methodological quality assessment in duplicate. A pre-piloted standardized data extraction form was utilized to record key information, including study characteristics, trial eligibility criteria, baseline demographic and clinical profiles, full details of intervention and comparator regimens, outcome definitions and assessment time points, follow-up duration, and funding sources, in addition to the predefined outcome measures. Any discrepancies between the two reviewers were resolved through discussion to reach a consensus, with arbitration by a third reviewer (W.S.) when necessary.

The risk of bias for the included trials was evaluated using the Cochrane Risk of Bias 2 (RoB 2) tool (9), the current validated standard for randomized controlled trials. This instrument assesses bias across five core domains: (i) randomization process; (ii) deviations from intended interventions; (iii) missing outcome data; (iv) measurement of the outcome; and (v) selection of the reported result. Each domain was independently categorized as having “low risk of bias,” “some concerns,” or “high risk of bias,” and an overall risk of bias judgement was derived for each study based on the domain-level evaluations.

### Data analysis

Statistical analyses were performed using Review Manager (Revman) version 5.4 (The Cochrane Collaboration, Copenhagen, Denmark) and Stata17.0 (StataCorp, College Station, TX, USA). For dichotomous outcomes, treatment effects comparing short-course versus long-course antibiotic therapy were expressed as pooled risk ratios (RRs) with the corresponding 95% confidence intervals (CIs). The Mantel–Haenszel method was used for fixed-effects models, and the inverse variance method with the DerSimonian–Laird estimator was adopted for random-effects models.

Statistical heterogeneity among the included studies was assessed using Cochrane’s Q test (with *p* < 0.10 considered statistically significant for heterogeneity) and quantified by the *I*^2^ statistic, where *I*^2^ values of 25, 50, and 75% correspond to low, moderate, and high levels of heterogeneity, respectively. A fixed-effects model was applied when heterogeneity was low to moderate (*I*^2^ ≤ 50%). Conversely, when substantial heterogeneity was detected (*I*^2^ > 50%), a random-effects model (DerSimonian-Laird method) was adopted to generate more conservative estimates; we further performed leave-one-out sensitivity analysis to identify potential outlier studies and to explore potential sources of heterogeneity. Overall statistical significance for the pooled effects was established at a two-sided *p* < 0.05.

To evaluate the robustness of the pooled results, a prespecified leave-one-out sensitivity analysis was conducted for outcomes with moderate to substantial heterogeneity. Each study was sequentially omitted to recalculate the pooled estimate, in order to verify the stability of the overall result and identify studies that disproportionately contributed to between-study heterogeneity.

Prespecified subgroup analyses stratified by antibiotic class were performed to explore the consistency of treatment effects across different antimicrobial regimens, including macrolides, fluoroquinolones, β-lactams, and pleuromutilins (lefamulin). Between-subgroup differences were tested using the Cochrane’s Q-based chi-square test to determine whether antibiotic class was a potential effect modifier.

Furthermore, to evaluate the potential presence of publication bias for the primary outcome, funnel plot visual inspection supplemented by Begg’s rank correlation test and Egger’s linear regression test was performed, provided that at least 10 studies were available. A *p*-value > 0.05 in these quantitative tests was interpreted as no statistically significant evidence of publication bias. Duval and Tweedie trim-and-fill analysis (10) was additionally conducted to further assess the impact of potential unpublished studies on the pooled effect estimate. For outcomes with fewer than 10 included studies, formal publication bias testing was not performed due to limited statistical power.

## Results

**Figure 1:**
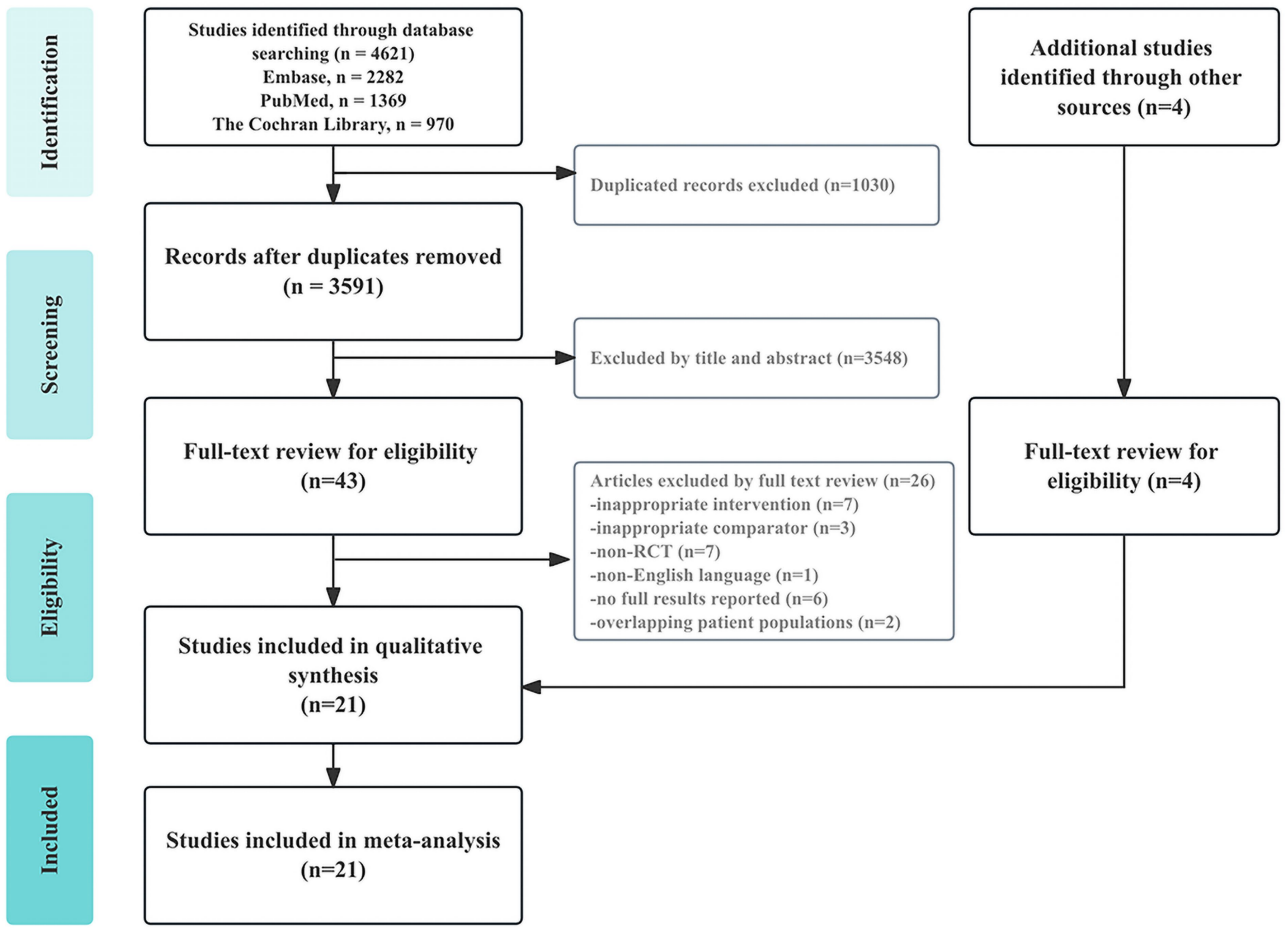
Study flowchart.

A total of 4,621 records were initially retrieved from PubMed, Embase, and CENTRAL databases, and 4 additional records were identified through manual screening of reference lists. After removal of 1,030 duplicates, 3,591 unique records remained for title and abstract screening. Of these, 3,548 records were excluded based on pre-defined eligibility criteria, leaving 43 full-text articles for detailed assessment. After full-text review, 21 RCTs met all inclusion criteria and were included in the final systematic review and meta-analysis (7, 11–30). The detailed study selection process and reasons for exclusion are presented in the PRISMA flowchart (Figure 1).

A total of 5,812 participants were included in the meta-analysis, with 2,939 in the short-course group and 2,873 in the long-course group. The included trials were conducted across North America, Europe, Asia, Africa, and South America, with study designs including double-blind, open-label, and double-dummy non-inferiority trials. All study populations comprised adult patients aged 18 to 94 years with a confirmed diagnosis of community-acquired pneumonia. The duration of short-course regimens ranged from 3 to 5 days, while long-course regimens ranged from 7 to 14 days. The included antibiotics covered four main classes: macrolides, fluoroquinolones, *β*-lactams, and pleuromutilins (lefamulin), which formed the basis for our prespecified subgroup analyses. The baseline demographic and clinical characteristics of the included studies are summarized in Table 1.

**Table 1:** Characteristics of the included studies.

First author, year	Study design	Country	Sex (male/female)		Age (years)		Pneumonia severity (PORT/PSI/CURB-65)		Regimen	
			Short-course	Long-course	Short-course	Long-course	Short course	Long course	Short course	Long course
Alexander, 2019 (11)	Double-blind, double-dummy, randomized non-inferiority clinical trial	19 countries (Europe, North America, South America, Asia, Africa)	207/163	180/188	57.4 ± 16.4	57.7 ± 16.2	I: 0.3% II: 49.5% III: 39.2% IV: 10.8% V: 0.3%	I: 0.5% II: 51.4% III: 36.1% IV: 1.1% V: 4.4%	Lefamulin 600 mg oral every 12 h for 5 days	Moxifloxacin 400 mg oral once daily for 7 days
Balmes, 1991 (12)	Open, comparative, multicentre, randomized clinical trial	France	30/22	39/19	57.9 (range: 19–94)	60.7 (rang: 22–83)	-	-	Azithromycin 500 mg on day 1, then 250 mg once daily on days 2–5	Amoxicillin/clavulanic acid 625 mg (amoxicillin 500 mg + clavulanic acid 125 mg) three times daily for 10 days
Barrera, 2016 (13)	Global, double-blind, double-dummy, randomised, active-controlled, non-inferiority trial	North America, Latin America, Europe, and South Africa (114 centres)	227/199	229/205	58.5 ± 14.7	56.7 ± 15.5	I: <1% II: 49% III: 39% IV: 11%	I: 0 II: 51% III: 40% IV: 9%	Oral solithromycin: 800 mg on day 1, 400 mg on days 2–5, placebo on days 6–7	Oral moxifloxacin: 400 mg once daily on days 1–7
Bohte, 1995 (14)	Randomized clinical trial	Netherlands	28/26	11/10	Pneumococcal group: 56 ± 14; Non-pneumococcal group: 51 ± 17	54 ± 17	-	-	Azithromycin 500 mg oral twice on the first day and once daily during the next 4 days	Erythromycin base was given orally for 10 days in a dose of 500 mg four times a day
Dinh, 2021 (7)	Double-blind, randomised, placebo-controlled, non-inferiority clinical trial	France	86/66	94/57	72.5 (range: 54.0–85.3)	74.0 (range: 58.0–83.0)	II: 37% III: 26% IV: 30% V: 8%	II: 36% III: 23% IV: 37% V: 4%	After 3 days of treatment with β-lactam therapy, patients matched placebo for 5 days	After 3 days of treatment with β-lactam therapy, patients received two pills of 500 mg of amoxicillin plus 62.5 mg of clavulanate orally three times a day for 5 days
D’Ignazio, 2005 (15)	Randomized, double-blind, double-dummy study utilized anoninferiority clinical trial	Canada	121/90	109/103	48.2 ± 18.1	49.0 ± 18.6	only enrolled patients with mild to moderate pneumonia categorized as Fine I-III	only enrolled patients with mild to moderate pneumonia categorized as Fine I-III	Single 2.0-g oral dose of azithromycin micro spheres and 7 days of once-daily levofloxacin placebo	7-day regimen of levofloxacin, 500 mg daily, and a single dose of azithromycin placebo
Drehobl, 2005 (16)	Multicenter, double-blind, double-dummy, randomized clinical trial	United States, Canada, Argentina, Russia, India, Estonia, Lithuania	112/135	134/118	45.6	43.6	only enrolled patients with Fine I-II	only enrolled patients with Fine I-II	A single 2.0-g dose of azithromycin microspheres on day 1, then two capsules placebo daily on days 2–7	Extended-release clarithromycin 1.0 g once daily (two 500 mg capsules once daily) for 7 days
Dunber, 2003 (17)	Randomized, double-blind, active treatment–controlled, noninferiority study	United States	148/108	162/110	53.1 ± 17.5	53.1 ± 17.5	I/II: 60.5% III/IV: 39.5%	I/II: 54.8% III/IV: 45.2%	Levofloxacin 750 mg once daily for 5 days	Levofloxacin 500 mg once daily for 10 days
el Moussaoui, 2006 (18)	Randomised, double blind, placebo controlled non-inferiority	Netherlands	34/22	37/26	54	60	I: 13% II: 46% III: 30% IV: 11%	I: 18% II: 41% III: 27% IV: 14%	Initial 3 days’ intravenous amoxicillin, oral placebo three times daily for 5 days	Initial 3 days’ intravenous amoxicillin, oral amoxicillin 750 mg three times daily for 5 days
File, 2007 (19)	Randomized, double-blind, active controlled, parallel-group study	Bulgaria, Croatia, Czech Republic, Lithuania, Poland, Romania, Russia, Ukraine, the USA	146/110	148/106	44.9 ± 16.4	45.9 ± 17.3	I: 61.7% II: 28.1% III: 8.6% IV: 1.2% V: 0.4%	I: 53.5% II: 33.5% III: 8.3% IV: 4.3% V: 0.4%	Oral gemifloxacin 320 mg once daily for 5 days	Oral gemifloxacin 320 mg once daily for 7 days
Kinasewitz, 1991 (20)	Randomised, double-blind study	United States	33/20	42/24	43.3 (range: 16–75)	40.2 (range: 16–83)	-	-	Azithromycin 500 mg single dose on day 1, followed by 250 mg once daily on days 2–5	Cefaclor 500 mg third daily for 10 days
O’Doherty, 1998 (21)	Randomized, multicentre, open-label, parallel-group study	Ireland	60/41	59/43	50.1 (range: 14.1–75.2)	51.5 (range: 12.5–78.9)	-	-	Azithromycin 500 mg once daily for 3 consecutive days	Clarithromycin 250 mg twice daily for 10 consecutive days
Paris, 2008 (22)	Randomised, open-label, non-inferiority study	Italy	70/66	80/51	42.4 ± 12.5	42.5 ± 13.1	I: 67.6% II: 32.4%	I: 67.2% II: 32.8%	Azithromycin 1 g (two 500 mg tablets) once daily for 3 days	Amoxicillin-Clavulanate 875/125 mg twice daily for 7 days
Rahav, 2004 (23)	Prospective, open-label, randomized, multicentre comparative study	Israel	25/37	28/18	50 ± 20	51 ± 18	-	-	Azithromycin 500 mg once daily for 3 days	other antibiotic therapy to treat pneumonia for 10 days
Rizzato, 1995 (24)	Open, randomized pilot study	Italy	13/7	16/4	48 ± 13	44 ± 19	-	-	Azithromycin 500 mg once daily for 3 days	Clarithromycin 250 mg twice daily for 10 ± 2 days (minimum 8 days)
Schönwald, 1990 (25)	Open, randomized, multicentre study	Yugoslavia	27/30	17/27	range: 12–79	range: 20–80	-	-	Azithromycin 250 mg twice daily on day 1, then 250 mg once daily on days 2–5	Erythromycin 500 mg four times daily for 10 days
Schönwald, 1994 (26)	Open, randomized, multicentre study	Croatia	49/40	32/21	38 ± 14	41 ± 11	-	-	Azithromycin 500 mg once daily for 3 days	Roxithromycin 150 mg twice daily for 10 days
Sopena, 2004 (27)	Prospective, open-label, randomized study	Spain	n = 34, Number of genders not listed	n = 36, Number of genders not listed	41.7	44.4	only enrolled patients with mild to moderate CAP	only enrolled patients with mild to moderate CAP	Azithromycin 500 mg once daily for 3 days	Clarithromycin 250 mg twice daily for 10–14 days
Tellier, 2004 (28)	Multicenter, randomized, double-blind, active-controlled study	Argentina	118/69	94/87	range: 18–79	range: 15–88	I: 49.2% II: 32.1% III: 12.3% IV: 6.4% V: 0	I: 48.1% II: 30.9% III: 15.5% IV: 5.5% V: 0	Telithromycin 800 mg once daily for 5 days	Clarithromycin 500 mg twice daily for 10 days
Zhao, 2014 (29)	Multicenter, randomized, open-label, non-inferiority controlled clinical trial	China	57/54	67/45	42.1 ± 16.4	39.6 ± 16.6	-	-	Levofloxacin 750 mg intravenous infusion once daily for 5 days	Levofloxacin 500 mg intravenous infusion once daily for 7–14 days
Zhao, 2016 (30)	Randomized, open, multicenter clinical study	China	112/109	108/119	39.43 ± 17.02	42.70 ± 16.81	0: 87.33% 1 point: 11.31% 2 points: 1.36%	0: 86.34% 1point: 11.45% 2 points: 2.2%	Levofloxacin 750 mg intravenous infusion once daily for 5 days	Levofloxacin 500 mg intravenous or oral sequential therapy once daily for 7–14 days

### Primary outcome

**Figure 2:**
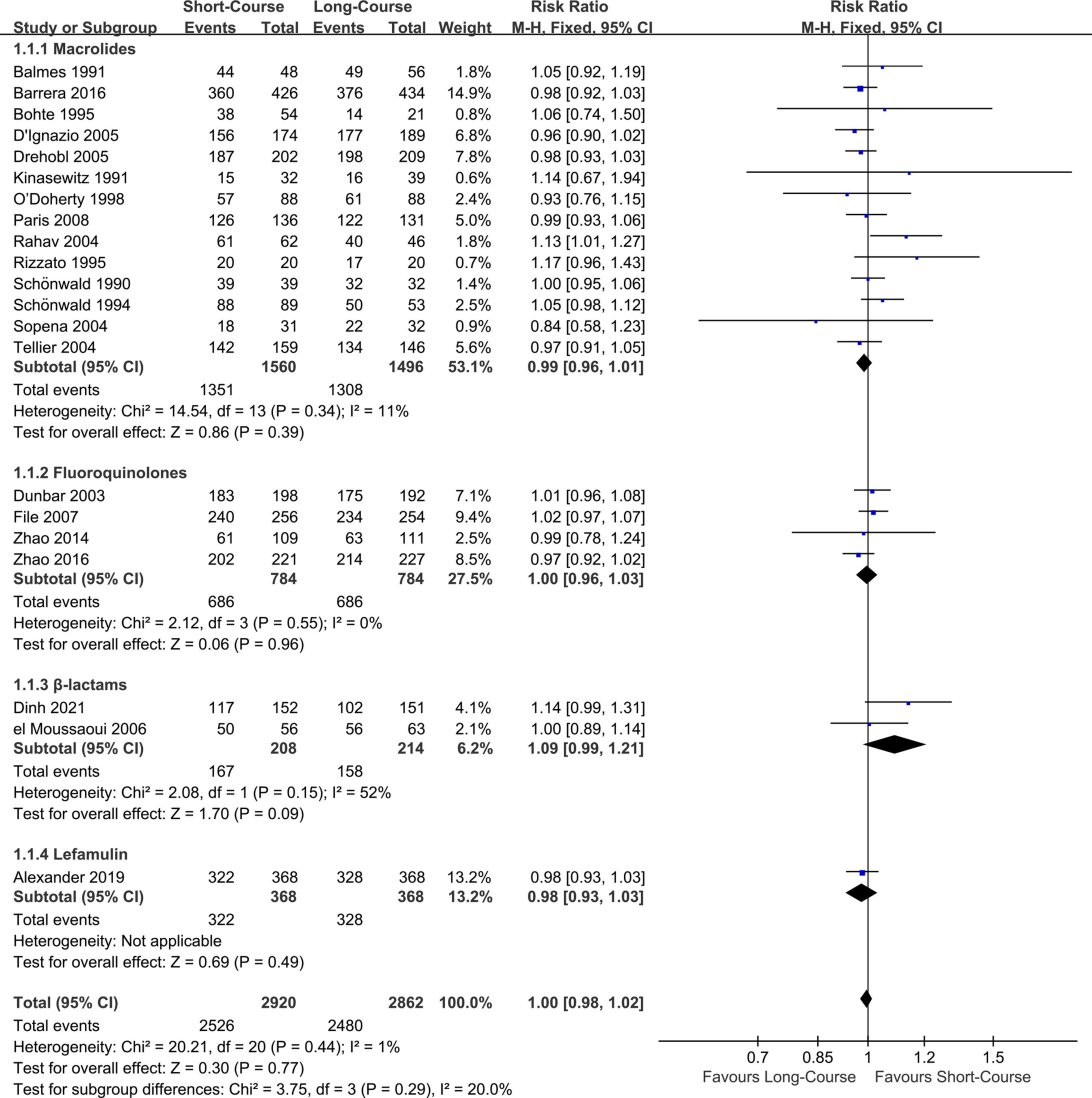
Forest plot depicting the risk ratios of clinical success for clinically evaluable patients receiving antibiotic treatment for short-course versus long-course regimen in the treatment of CAP.

For the primary outcome of clinical success, 21 RCTs were included in the quantitative meta-analysis, comparing the efficacy of short-course versus long-course antibiotic therapy in patients with CAP (Figure 2).

Statistical heterogeneity across the included studies was minimal (*χ*^2^ = 20.21, df = 20, *p* = 0.44; *I*^2^ = 1%). Accordingly, the Mantel–Haenszel fixed-effects model was employed to calculate the pooled risk ratio (RR) with 95% CIs. The pooled analysis demonstrated no statistically significant difference in clinical success between short-course and long-course antibiotic therapy (RR = 1.00, 95% CI: 0.98–1.02, *Z* = 0.30, *p* = 0.77). The majority of individual studies had 95% CIs crossing the null value of 1, which was consistent with the pooled estimate and confirmed comparable clinical efficacy between the two treatment regimens.

In prespecified subgroup analyses stratified by antibiotic class, no significant difference in treatment effect was detected across the four antimicrobial categories (macrolides, fluoroquinolones, *β*-lactams, and lefamulin) (test for subgroup differences: *χ*^2^ = 3.75, df = 3, *p* = 0.29, *I*^2^ = 20.0%), indicating that the consistency of efficacy was not modified by antibiotic class.

## Secondary outcomes

### Adverse events

**Figure 3:**
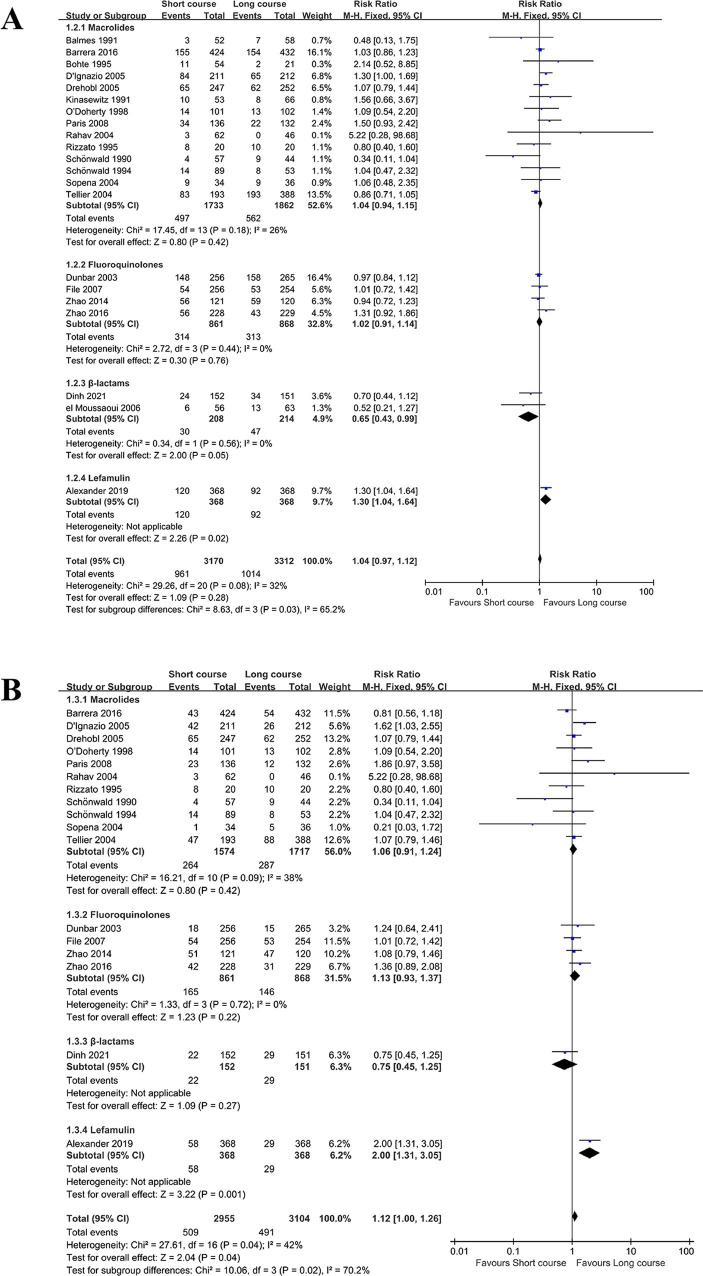
Forest plot depicting the risk ratios of adverse events for clinically evaluable patients receiving antibiotic treatment for short-course versus long-course regimen in the treatment of CAP. **(A)** Total AEs. **(B)** Treatment-related AEs.

For the outcome of AEs, 21 RCTs were included in the pooled analysis (Figure 3A). Moderate statistical heterogeneity was detected (*χ*^2^ = 29.26, df = 20, *p* = 0.08; *I*^2^ = 32%), and the Mantel–Haenszel fixed-effects model was applied. The pooled estimate showed no statistically significant difference in the risk of total AEs between short-course and long-course antibiotic therapy (RR = 1.04, 95% CI: 0.97–1.12; *Z* = 1.09, *p* = 0.28). In prespecified subgroup analyses stratified by antibiotic class, a statistically significant difference in treatment effect was observed across subgroups (test for subgroup differences: *χ*^2^ = 8.63, df = 3, *p* = 0.03, *I*^2^ = 65.2%). The elevated risk was mainly driven by the lefamulin subgroup, while no significant between-group difference was found in the macrolide, fluoroquinolone, and β-lactam subgroups.

For treatment-related AEs, pooled data from 17 RCTs were analyzed (Figure 3B). Moderate heterogeneity was detected (*χ*^2^ = 27.61, df = 16, *p* = 0.04; *I*^2^ = 42%). The fixed-effects model showed that short-course therapy was associated with a statistically significantly higher risk of treatment-related AEs compared with long-course therapy (RR = 1.12, 95% CI: 1.00–1.26; Z = 2.04, *p* = 0.04). Subgroup analyses revealed significant differences in effect sizes across antibiotic classes (test for subgroup differences: *χ*^2^ = 10.06, df = 3, *p* = 0.02, *I*^2^ = 70.2%), and the increased risk was predominantly driven by the lefamulin subgroup; no significant between-group difference was observed in the fluoroquinolone and β-lactam subgroups.

**Figure 4:**
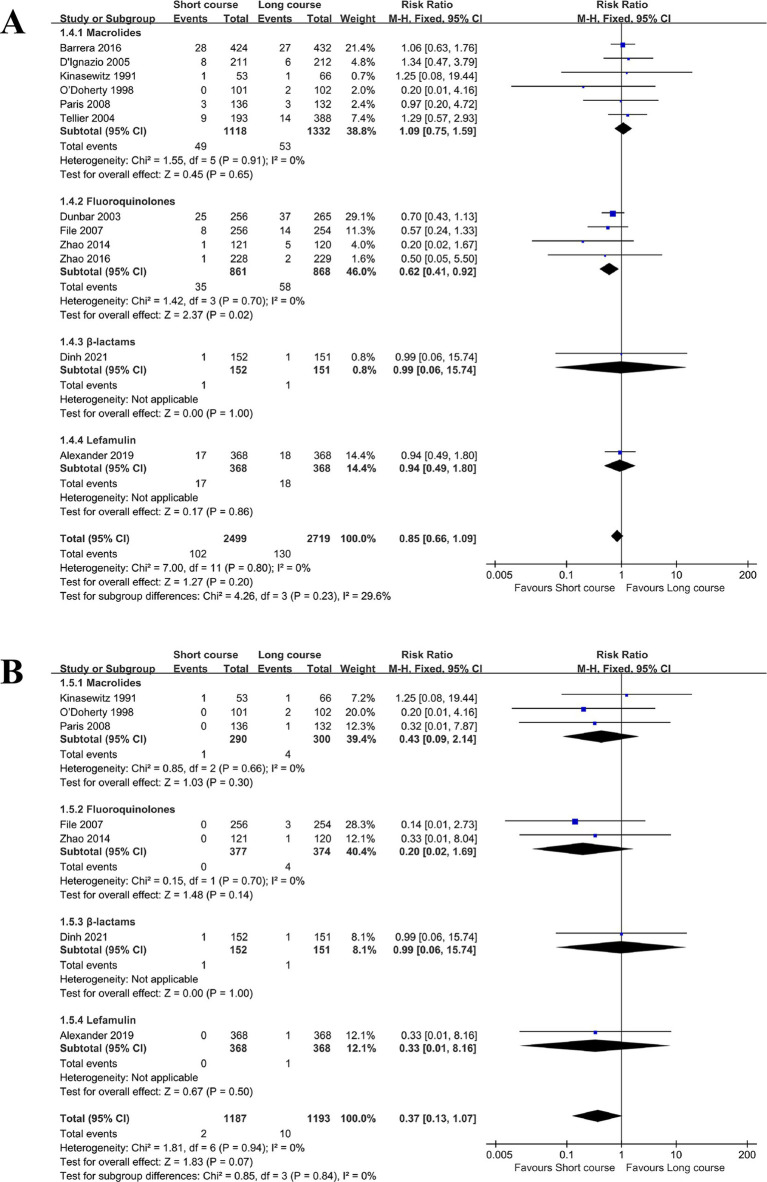
Forest plot depicting the risk ratios of serious adverse events for clinically evaluable patients receiving antibiotic treatment for short-course versus long-course regimen in the treatment of CAP. **(A)** Total SAEs. **(B)** Treatment-related SAEs.

For serious adverse events (SAEs), we separately analyzed total SAEs and treatment-related SAEs. The analysis of total SAEs included 12 RCTs (Figure 4A). No significant heterogeneity was observed (*χ*^2^ = 7.00, df = 11, *p* = 0.80; *I*^2^ = 0%). The fixed-effects model showed no statistically significant difference in the risk of total SAEs between the two groups (RR = 0.85, 95% CI: 0.66–1.09; *Z* = 1.27, *p* = 0.20). No significant difference was detected across antibiotic class subgroups (test for subgroup differences: *χ*^2^ = 4.26, df = 3, *p* = 0.23, *I*^2^ = 29.6%).

For treatment-related SAEs, 7 RCTs were analyzed. Only 2 events were reported in the short-course group versus 10 in the long-course group. With no significant heterogeneity (*χ*^2^ = 1.81, df = 6, *p* = 0.94; *I*^2^ = 0%), the fixed-effects model showed that short-course therapy was associated with a non-statistically significant trend toward reduced risk of treatment-related SAEs (RR = 0.37, 95% CI: 0.13–1.07; *Z* = 1.83, *p* = 0.07). Subgroup differences across antibiotic classes were not statistically significant (test for subgroup differences: *χ*^2^ = 0.85, df = 3, *p* = 0.84, *I*^2^ = 0%).

### Bacteriological success

**Figure 5:**
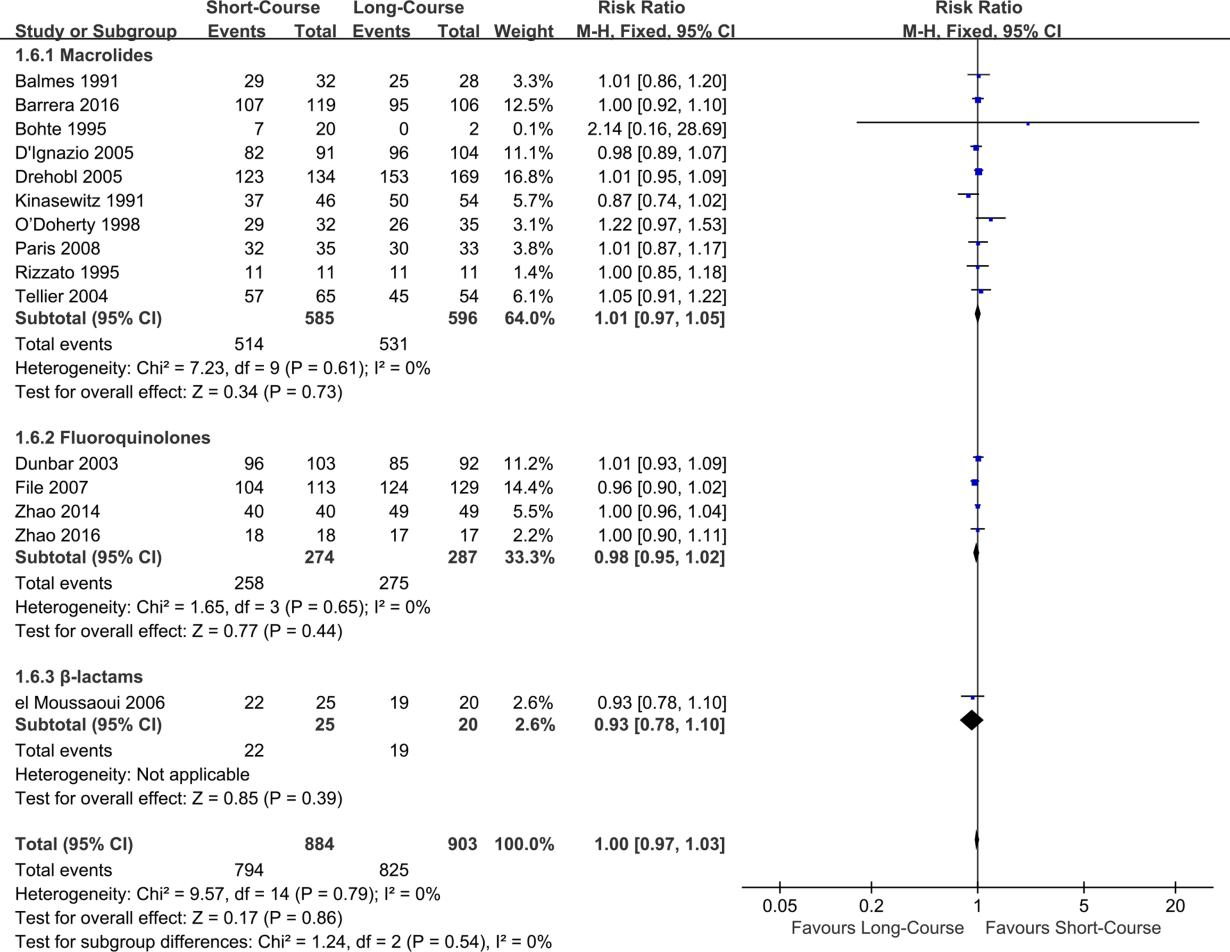
Forest plot depicting the risk ratios of bacteriological success for clinically evaluable patients receiving antibiotic treatment for short-course versus long-course regimen in the treatment of CAP.

Bacteriological success, a key secondary outcome assessing microbiological efficacy, was evaluated in 15 RCTs (Figure 5). Successful bacteriological response was documented in 794 patients in the short-course group and 825 patients in the long-course group. No significant heterogeneity was detected across studies (*χ*^2^ = 9.57, df = 14, *p* = 0.79; *I*^2^ = 0%), and a fixed-effects model was applied. The pooled analysis revealed no significant difference in bacteriological success rates between the two regimens (RR = 1.00, 95% CI: 0.97–1.03; *Z* = 0.17, *p* = 0.86). This finding was consistent with the majority of individual studies, whose confidence intervals crossed the null value of 1, confirming comparable microbiological efficacy between short-course and long-course antibiotic therapy for CAP.

In prespecified subgroup analyses stratified by antibiotic class, no significant difference in treatment effect was identified across the macrolide, fluoroquinolone, and *β*-lactam subgroups (test for subgroup differences: *χ*^2^ = 1.24, df = 2, *p* = 0.54, *I*^2^ = 0%). This indicated that the comparable microbiological efficacy of short-course versus long-course therapy was consistent across different antimicrobial categories.

### Radiological success

**Figure 6:**
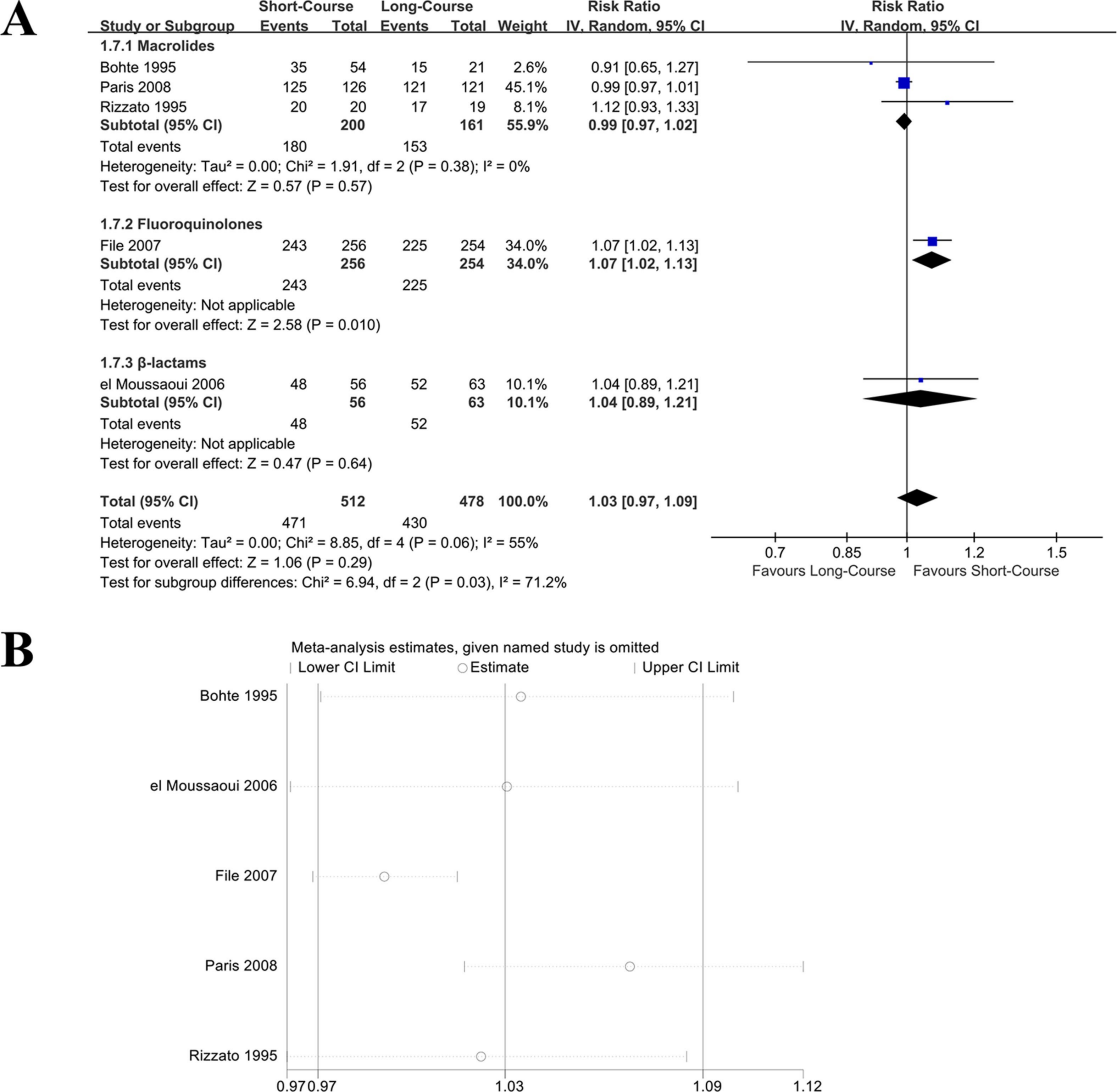
Radiological success of short-course versus long-course antibiotic therapy for CAP. **(A)** Forest plot of pooled risk ratios using a random-effects model. **(B)** Leave-one-out sensitivity analysis plot, showing the pooled risk ratio and 95% confidence interval after sequential omission of each included study.

Radiological success, another key secondary endpoint reflecting treatment efficacy in imaging improvement, was assessed across 5 included studies (Figure 6A). A total of 512 patients in the short-course group and 478 patients in the long-course group were analyzed, with 471 and 430 events of radiological success achieved, respectively.

Moderate statistical heterogeneity was detected across studies (Tau^2^ = 0.00; *χ*^2^ = 8.85, df = 4, *p* = 0.06; *I*^2^ = 55%), so the inverse variance random-effects model was applied for data synthesis. The pooled results demonstrated no statistically significant difference in radiological success rates between the short-course and long-course antibiotic regimens (RR = 1.03, 95% CI 0.97–1.09; *Z* = 1.06, *p* = 0.29).

In prespecified subgroup analyses stratified by antibiotic class, a statistically significant difference in treatment effect was identified across the macrolide, fluoroquinolone, and *β*-lactam subgroups (test for subgroup differences: *χ*^2^ = 6.94, df = 2, *p* = 0.03, *I*^2^ = 71.2%). Specifically, the fluoroquinolone subgroup showed a significantly higher radiological success rate in the short-course regimen (RR = 1.07, 95% CI 1.02–1.13; *p* = 0.010), while no significant differences were observed in the macrolide (RR = 0.99, 95% CI 0.97–1.02; *p* = 0.57) and *β*-lactam subgroups (RR = 1.04, 95% CI 0.89–1.21; *p* = 0.64).

To systematically explore the sources of heterogeneity and verify the robustness of the pooled estimate, a leave-one-out sensitivity analysis was performed, with full results presented in Supplementary Table S1. After sequential omission of each individual study, the pooled risk ratios remained generally stable, ranging from 1.00 to 1.06. Two core findings were confirmed: first, after omitting the study by Paris et al. (22), the between-study heterogeneity decreased from 55 to 0% (Tau^2^ = 0.00), verifying that this study was the primary contributor to the overall heterogeneity, mainly due to its near-ceiling radiological response rate and distinct imaging assessment protocol. Second, the statistical significance of the pooled effect was completely abolished after omitting the study by File et al. (19) (RR = 1.00, 95% CI 0.95–1.06; *p* = 0.86), indicating that the trend of short-course superiority in radiological success was predominantly driven by this single fluoroquinolone trial.

### Mortality

**Figure 7:**
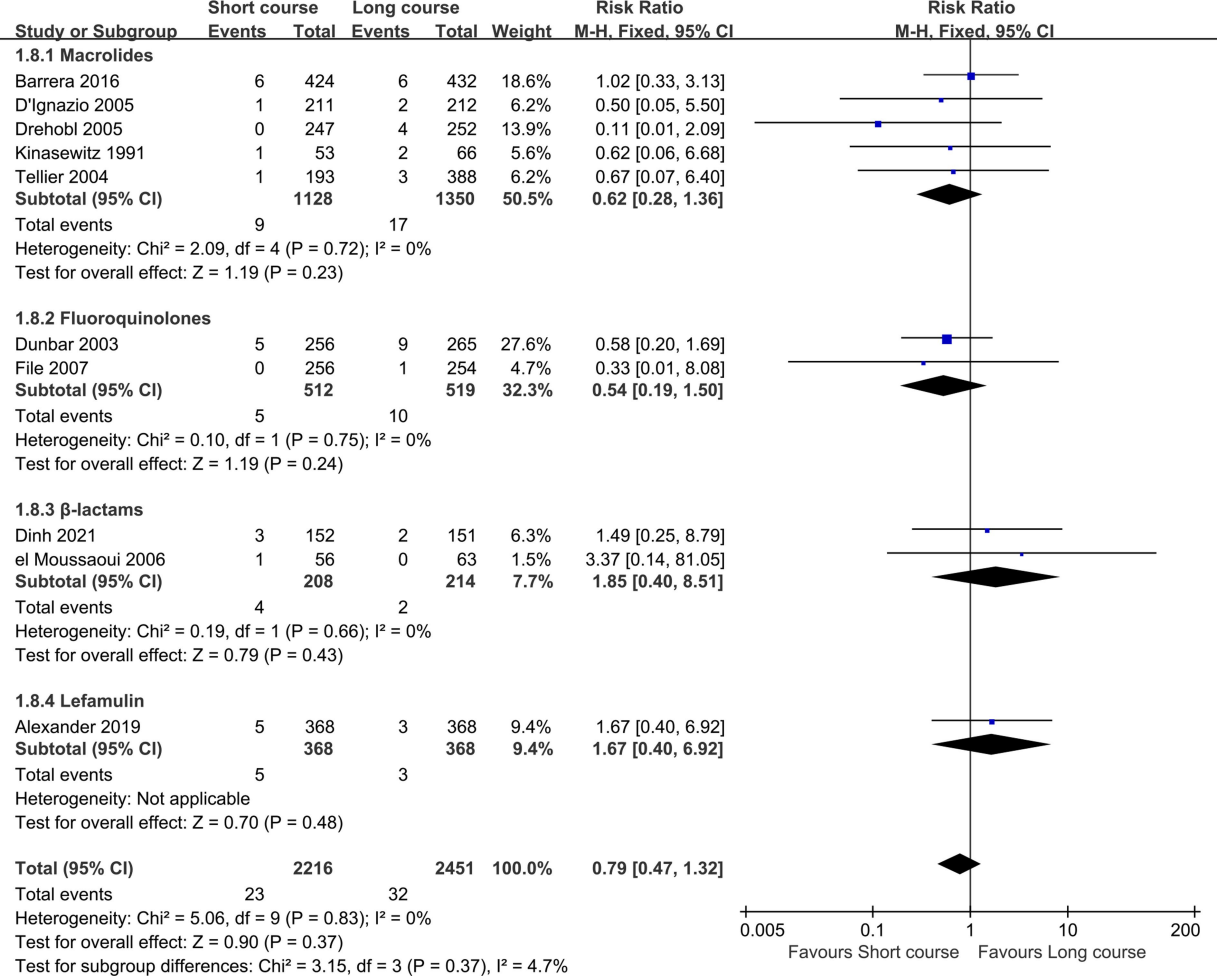
Forest plot depicting the risk ratios of mortality for clinically evaluable patients receiving antibiotic treatment for short-course versus long-course regimen in the treatment of CAP.

All-cause mortality was evaluated in 10 RCTs comprising 2,216 patients in the short-course group (23 events) and 2,451 in the long-course group (32 events) (Figure 7). No significant heterogeneity was detected across studies (*χ*^2^ = 5.06, df = 9, *p* = 0.83; *I*^2^ = 0%), and a fixed-effects model was applied. The pooled analysis yielded a risk ratio of 0.79 (95% CI: 0.47–1.32, *Z* = 0.90, *p* = 0.37), indicating no statistically significant difference in mortality risk between short-course and long-course antibiotic regimens for CAP patients.

In prespecified subgroup analyses stratified by antibiotic class, no significant difference in treatment effect was identified across the macrolide, fluoroquinolone, β-lactam, and lefamulin subgroups (test for subgroup differences: *χ*^2^ = 3.15, df = 3, *p* = 0.37, *I*^2^ = 4.7%), indicating that the comparable mortality risk between the two regimens was consistent across different antimicrobial categories.

### Discontinuation

**Figure 8:**
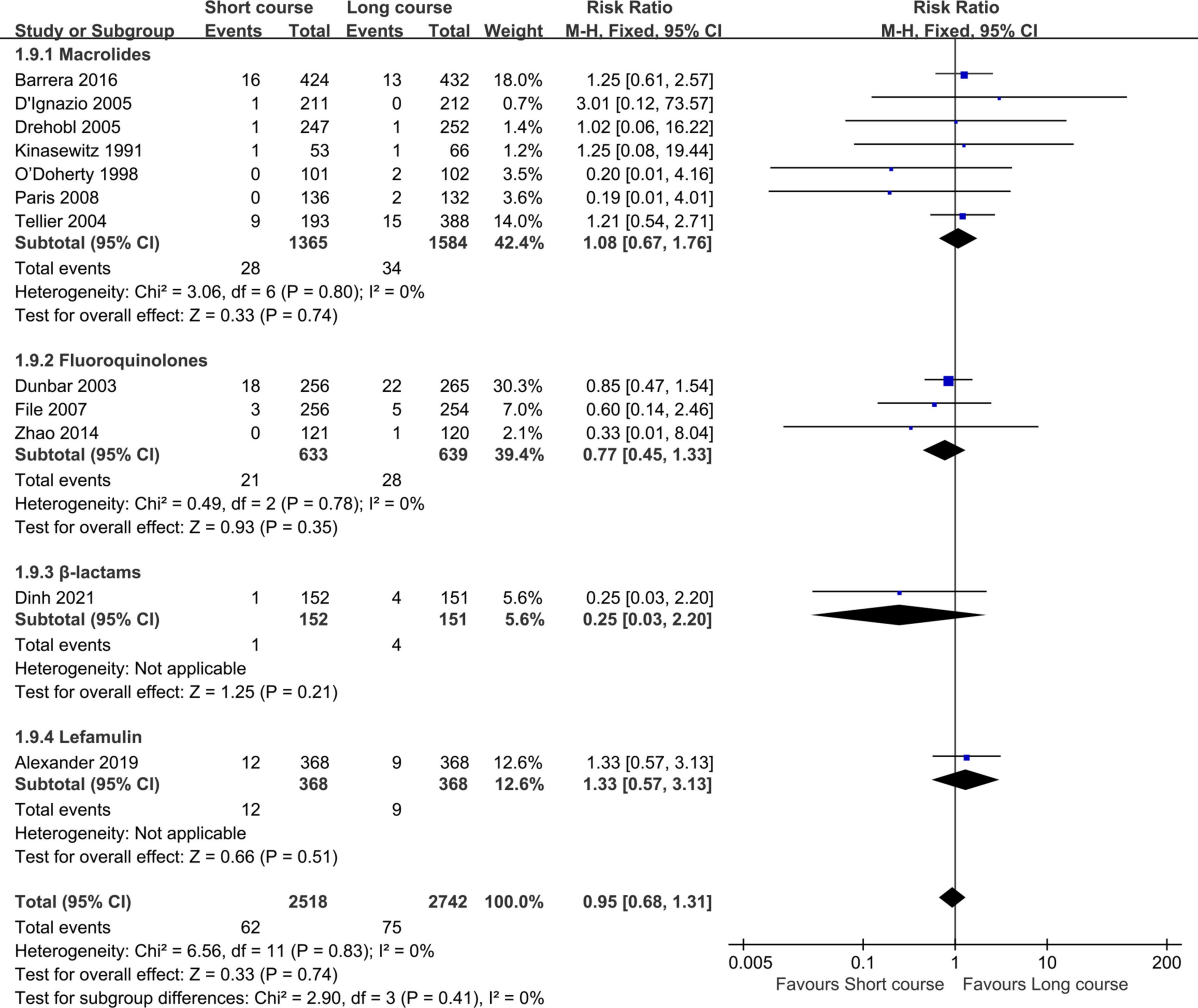
Forest plot depicting the risk ratios of discontinuation for clinically evaluable patients receiving antibiotic treatment for short-course versus long-course regimen in the treatment of CAP.

Treatment discontinuation, a secondary outcome reflecting tolerability, was analyzed in 12 RCTs comparing short-course and long-course antibiotic therapy. The short-course group included 2,518 patients (62 events) and the long-course group 2,742 patients (75 events) (Figure 8). No significant heterogeneity was detected across studies (*χ*^2^ = 6.56, df = 11, *p* = 0.83; *I*^2^ = 0%), and a fixed-effects model was applied. The pooled analysis revealed no significant difference in treatment discontinuation rates between the two regimens (RR = 0.95, 95% CI: 0.68–1.31, *Z* = 0.33, *p* = 0.74), with the confidence interval crossing the null value of 1, indicating comparable treatment tolerability between short-course and long-course therapy for patients with CAP.

In prespecified subgroup analyses stratified by antibiotic class, no significant difference in treatment effect was identified across the macrolide, fluoroquinolone, *β*-lactam, and lefamulin subgroups (test for subgroup differences: *χ*^2^ = 2.90, df = 3, *p* = 0.41, *I*^2^ = 0%), indicating that the comparable tolerability between the two regimens was consistent across different antimicrobial categories.

### Quality assessment

**Figure 9:**
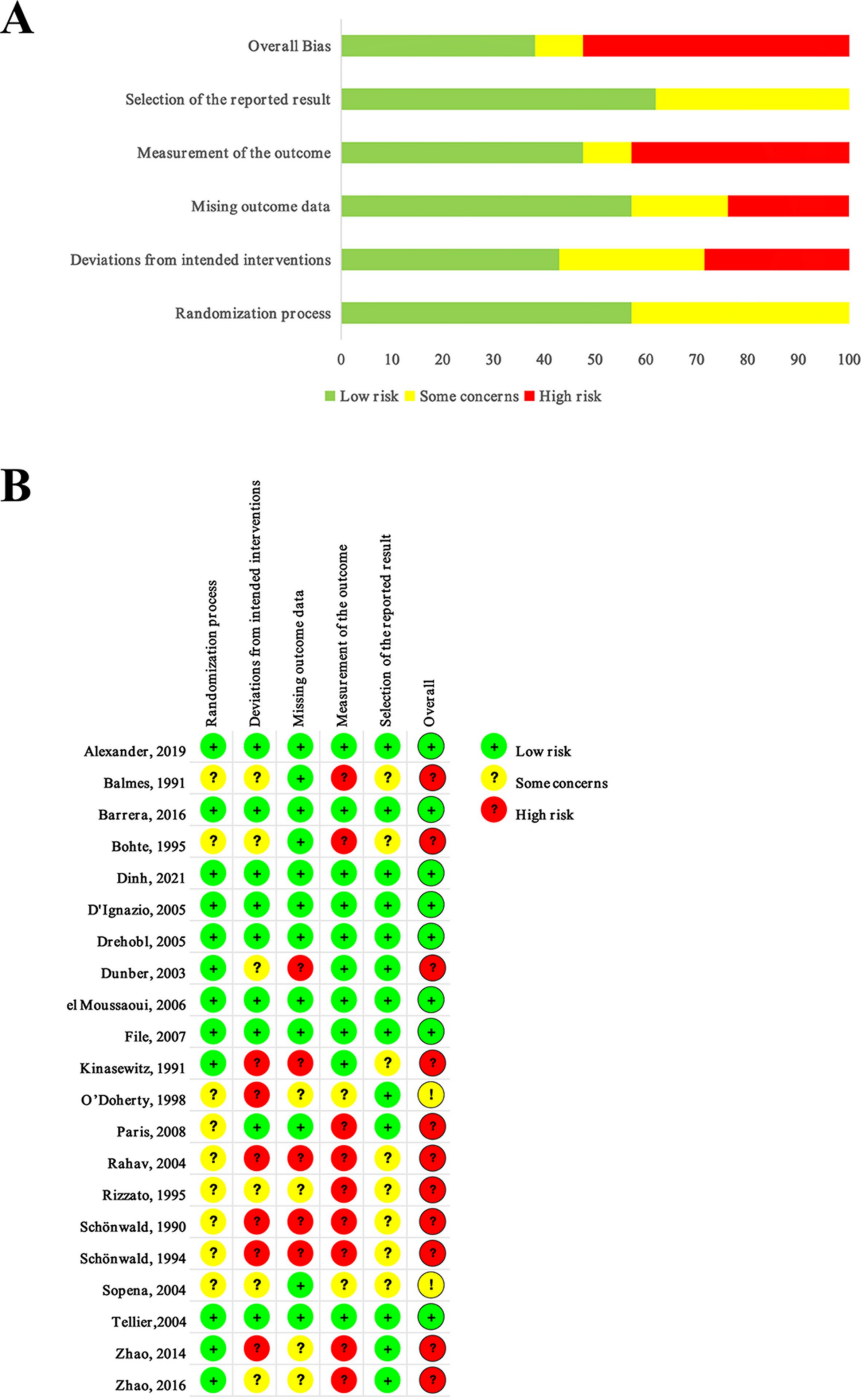
Risk of bias analysis and quality assessment of included trials. **(A)** Risk of bias graph: judgement of review authors about each risk of bias item presented as percentages across all included studies. **(B)** Risk of bias summary: judgement of review authors about each risk of bias item for each included study.

Methodological quality and risk of bias were systematically evaluated for all 21 included RCTs using the RoB2 tool, covering five core domains: randomization process, deviations from intended interventions, missing outcome data, measurement of the outcome, and selection of the reported result (Figure 9).

Overall, the included studies presented generally acceptable methodological quality. Most trials were judged to have low risk of bias in the domains of randomization process, missing outcome data, and selection of the reported result. Some concerns were sporadically observed across a subset of trials, predominantly related to performance bias and detection bias, which stemmed from the practical difficulty of implementing strict blinding in open-label antibiotic trial designs. A small number of studies were rated as having high risk of bias in individual domains, but no critical flaws that would fundamentally undermine the validity of pooled results were identified.

Collectively, the risk of bias assessment indicated that the included evidence had overall acceptable methodological reliability, supporting the robustness of the pooled findings in this meta-analysis.

### Publication bias

Publication bias for the primary outcome of clinical success was assessed via visual inspection of funnel plots, supplemented by quantitative methods including Egger’s linear regression test, Begg’s rank correlation test, and Duval and Tweedie trim-and-fill analysis.

**Figure 10:**
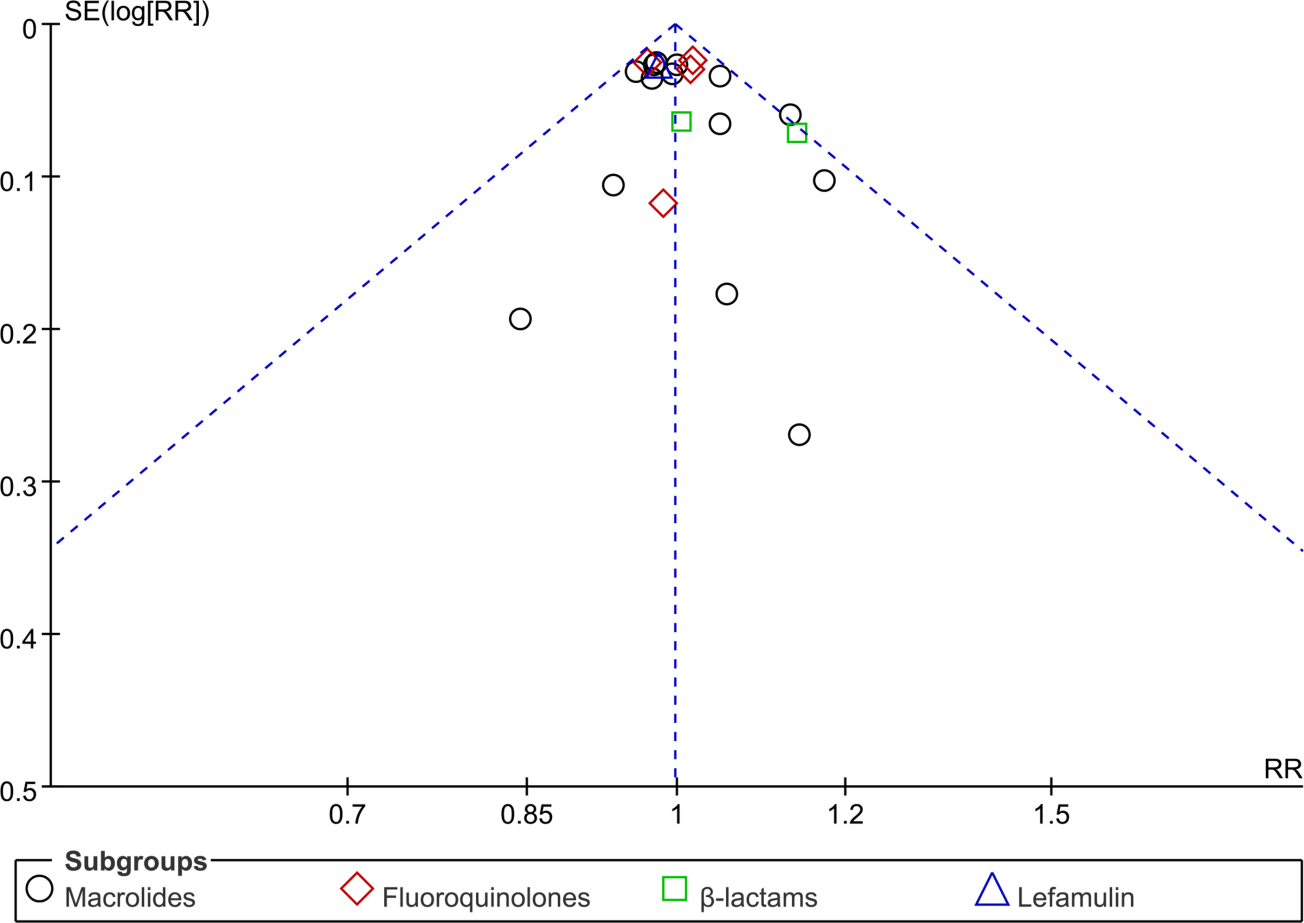
Funnel plot for publication bias of clinical success.

The funnel plot exhibited a generally symmetric distribution of individual study effect sizes, and no obvious asymmetry suggestive of significant publication bias was observed by visual assessment (Figure 10). Quantitative tests further supported this finding: Egger’s regression test showed no statistically significant intercept deviation (intercept = −0.027, *t* = 1.60, df = 19, *p* = 0.127), and Begg’s rank correlation test yielded a consistent non-significant result (*z* = 1.15, *p* = 0.251). Neither test detected statistically significant publication bias.

Additionally, the trim-and-fill sensitivity analysis was performed under the assumption that any funnel asymmetry was attributable to publication bias. The pooled effect size remained nearly unchanged after adjustment for potential missing studies (before trim-and-fill: RR = 1.00, 95% CI 0.98–1.02; after trim-and-fill: RR = 1.00, 95% CI 0.98–1.01), indicating that even if potential publication bias existed, it would not materially alter the pooled estimate of the primary outcome.

## Discussion

In this comprehensive systematic review and meta-analysis of 21 RCTs, we demonstrated that short-course antibiotic therapy (≤5 days) achieves comparable clinical efficacy relative to traditional long-course therapy (>5 days) for adults with CAP. The pooled analysis confirmed comparable rates of clinical success (RR = 1.00), bacteriological success, and all-cause mortality. Crucially, our study reveals a nuanced safety profile: while short-course therapy was associated with a marginally significant increase in treatment-related adverse events, treatment-related serious adverse events exhibited a non-significant downward trend.

These findings strongly reinforce the evidence-based trend toward shortened antibiotic duration that has increasingly dominated the infectious disease literature. The 2019 American Thoracic Society/Infectious Diseases Society of America (ATS/IDSA) guidelines advocate for a minimum of 5 days of therapy for CAP, provided the patient achieves clinical stability (31). Our data, representing one of the most comprehensive pooled analyses on this topic to date, not only validates this recommendation but suggests that ≤5 days is a safe and effective standard for clinically stable adult CAP patients. This aligns precisely with landmark trials, such as the PTCMA trial by Dinh et al. (7), which demonstrated the safety of discontinuing β-lactams after just 3 days in non-critical CAP patients, and classic studies by el Moussaoui et al. (18)

The most salient and novel contribution of our analysis is the subgroup-stratified elucidation of antibiotic safety. The observed dichotomy, higher general treatment-related AEs in the overall pooled analysis was predominantly driven by the lefamulin subgroup, while no significant between-group difference was detected in macrolides, fluoroquinolones, or *β*-lactams. This pattern is biologically plausible: to achieve rapid bactericidal effects, some short-course regimens employ higher daily doses or specific antimicrobial agents that can precipitate transient, dose-dependent side effects, such as gastrointestinal intolerance or nausea (32). Conversely, severe complications, such as *Clostridioides difficile* infection, severe organ toxicity, and profound microbiome disruption, are fundamentally driven by the cumulative duration of antibiotic exposure and prolonged ecological pressure (33). As Vaughn et al. demonstrated, each excess day of antibiotic therapy significantly increases the risk of patient harm without improving clinical outcomes (34). Therefore, the overall safety-benefit profile strongly favors short-course therapy for most patients: transient, self-limiting side effects are an acceptable compromise to prevent the cumulative risks of prolonged antibiotic exposure.

The consistent null effect across bacteriological and radiological outcomes further validates our clinical efficacy findings. The comparable bacteriological success rate effectively dispels the long-standing clinical concern that abbreviated therapy leads to inadequate pathogen eradication and subsequent relapse. While we observed moderate heterogeneity in radiological success (*I*^2^ = 55%), this is an expected phenomenon. Radiographic resolution notoriously lags behind clinical recovery, and the timing, protocols, and interpretations of follow-up imaging varied widely across the included trials (35). Therefore, delayed radiographic clearance should not preclude the cessation of antibiotics if the patient meets clinical stability criteria.

Despite its methodological rigor, this meta-analysis has several limitations. First, the definition, adjudication, and reporting of treatment-related AEs lacked strict standardization across the included trials, contributing to moderate heterogeneity (*I*^2^ = 42%). Second, while the reduction in treatment-related SAEs showed a favorable trend, it did not reach statistical significance, and the estimate was based on a low absolute event rate (2 events in the short-course group vs. 10 in the long-course group); thus, the exact magnitude of this protective effect warrants cautious interpretation. Future large-scale real-world studies are warranted to precisely quantify the true safety benefit of shortened regimens. Third, most included trials compared different antimicrobial agents across study arms in addition to different treatment durations, meaning the pooled effect sizes cannot be solely attributed to differences in treatment duration, and residual confounding by antibiotic class remains a notable methodological limitation. Although prespecified subgroup analyses stratified by antibiotic class showed a consistent direction of effect across all antimicrobial categories, which partially mitigates this concern, it cannot fully eliminate the confounding influence of varying antibiotic selection. Furthermore, clinical heterogeneity exists regarding disease severity and specific pathogens across trials, meaning these population-average results may still require tailoring for highly complex patients (e.g., those with *Pseudomonas aeruginosa* or severe immunocompromise). Fourth, the number of included studies for radiological success was limited, which restricted the statistical power of subgroup analyses for this endpoint; publication bias assessment was only performed for the primary outcome due to insufficient study numbers for secondary endpoints, and conventional tests have limited sensitivity for near-null effect sizes such that small-study effects cannot be fully excluded. Additionally, the restriction to English-language studies carries a potential risk of language bias, although the included trials cover multiple non-English-speaking regions, which partially mitigates this concern. Finally, most included primary studies lacked detailed subgroup stratification for specific pathogen subtypes or high-risk comorbid populations. Future research should focus on leveraging biomarkers in combination with rapid multiplex molecular diagnostics to deliver individualized, response-guided treatment duration strategies for patients with complicated severe CAP, thereby addressing current guideline gaps in the management of these special populations.

## Conclusion

Short-course antibiotic therapy (≤5 days) for adults with CAP achieves comparable efficacy across clinical, bacteriological, and survival endpoints relative to traditional extended courses, with a favorable trend toward reduced treatment-related serious adverse events rather than statistically significant superiority in severe safety outcomes. By demonstrating equivalent rates of clinical success, bacteriological success, and all-cause mortality, alongside a non-significant reduction in severe adverse events, this meta-analysis provides robust up-to-date evidence to support antibiotic duration shortening in clinically stable adult CAP patients, challenging the conventional practice of routine prolonged prescribing.

Adopting ≤5 days regimens for patients who achieve early clinical stability aligns closely with global antimicrobial stewardship priorities, maximizing therapeutic efficacy while mitigating the cumulative harms of prolonged antibiotic exposure, including antimicrobial resistance and severe drug toxicity. Clinicians may safely implement abbreviated therapy as a standard approach for appropriately selected adult CAP patients, and these findings provide high-quality supportive evidence to inform future guideline updates on optimized CAP treatment duration.

## Data Availability

The original contributions presented in the study are included in the article/Supplementary material, further inquiries can be directed to the corresponding author.
