# Supplementary material for: Efficacy and safety of short-course antibiotic therapy for community-acquired pneumonia in adults: a meta-analysis
Source: Front Med (Lausanne). 2026 Jul 15;13:1861027. doi: 10.3389/fmed.2026.1861027 (PMC13416331; doi:10.3389/fmed.2026.1861027)
Supplement: Supplementary file 1 [file Data_Sheet_1.docx]

Table S1 Results of leave-one-out sensitivity analysis for radiological success.

| Study omitted | Risk Ratio | Lower 95% CI | Upper 95% CI | *Z* value | *P* value | Tau² | *I*² (%) |
| --- | --- | --- | --- | --- | --- | --- | --- |
| Bohte 1995 | 1.05 | 1.01 | 1.08 | 2.48 | 0.013 | 0.001 | 70 |
| el Moussaoui 2006 | 1.04 | 1 | 1.08 | 2.03 | 0.042 | 0.001 | 70 |
| File 2007 | 1 | 0.95 | 1.06 | 0.17 | 0.862 | 0 | 0 |
| Paris 2008 | 1.06 | 1.01 | 1.11 | 2.16 | 0.031 | 0 | 0 |
| Rizzato 1995 | 1.04 | 1 | 1.08 | 1.81 | 0.07 | 0.001 | 63 |

Note: The analysis was performed based on the inverse variance random-effects model (DerSimonian–Laird method). Effect sizes are presented as risk ratios (RRs) with corresponding 95% confidence intervals (CIs). Tau² represents the estimated between-study variance, and *I*² indicates the proportion of total variation due to between-study heterogeneity.
